# Association Between Functional Limitations and Incident Cardiovascular Diseases and All-Cause Mortality Among the Middle-Aged and Older Adults in China: A Population-Based Prospective Cohort Study

**DOI:** 10.3389/fpubh.2022.751985

**Published:** 2022-02-11

**Authors:** Zhao Hu, Baohua Zheng, Atipatsa Chiwanda Kaminga, Feixiang Zhou, Huilan Xu

**Affiliations:** ^1^Department of Social Medicine and Health Management, Xiangya School of Public Health, Central South University, Changsha, China; ^2^Department of Epidemiology and Health Statistics, Xiangya School of Public Health, Central South University, Changsha, China; ^3^Department of Mathematics and Statistics, Mzuzu University, Luwinga, Mzuzu, Malawi

**Keywords:** functional limitation, cardiovascular diseases, elderly, cohort study, all-cause mortality

## Abstract

**Background:**

The prevalence of functional limitations is relatively high among the middle-aged and older adults. However, the contribution of functional limitations to subsequent incident cardiovascular diseases (CVD) and death is unclear. This study aims to examine the association between functional limitations and incident CVD and all-cause mortality among the middle-aged and older adults.

**Methods:**

This is a nationally representative prospective cohort study. Participants were middle-aged and older Chinese adults from The China Health and Retirement Longitudinal Study. Functional limitations were measured using activities of daily living (ADL) scale and instrumental activities of daily living (IADL) scale. Incident CVD and death were recorded at followed-up from June 1, 2011, up until August 31, 2018. Cox proportional hazards model was used to assess the association between functional limitations and incident CVD and all-cause mortality.

**Results:**

A total of 11,013 participants were included in this study. During the 7 years of follow-up, 1,914 incident CVD and 1,182 incident deaths were identified. Participants with functional limitations were associated with a 23% increased risk of incident CVD (HR, 1.23, 95% CI:1.08,1.39) after adjusting for age, gender, residential area, marital status, education, smoking, alcohol drinking, sleep duration, nap duration, depression symptoms, social participation, history of hypertension, diabetes, dyslipidemia, use of hypertension medications, diabetes medications, and lipid-lowering therapy. Moreover, participants with functional limitations were associated with a 63% increased risk of all-cause mortality (HR,1.63, 95%CI: 1.41,1.89) after adjusting for potential confounders.

**Conclusions:**

Functional limitations were significantly associated with subsequent incident CVD and death among the middle-aged and older Chinese adults.

## Introduction

Functional ability is an important indicator that reflects the quality of life and health status. With the population of middle-aged and older adults is increasing in China, the prevalence and incidence of functional limitations among this group is relatively high and posing significant medical challenges to the nation and care-givers (1, 2). A recent longitudinal study reported that 15.3 and 19.2% of the Chinese adults aged 45 and over developed functional limitations during 4 years of follow-up according to the definition of activities of daily living (ADL) scale and instrument activities of daily living (IADL) scale, respectively (3). Previous epidemiological studies demonstrated that cognitive function impairment, depression, unhealthy lifestyle behaviors, chronic conditions and multimorbidity were potential predictors of functional limitations (4–6). Furthermore, several studies found that functional limitations were associated with a range of adverse health outcomes and higher mortality risk (7, 8).

The incident cardiovascular diseases (CVD), well known for their heavy economic and social burden (9), seriously endanger health and quality of life (10). The prevalence of and mortality due to CVD in China is continuously rising (9, 11, 12). Specifically, CVD has been the leading cause of death in China, ahead of oncology and other diseases, accounting for 45.91 and 43.56% of deaths in Chinese rural and urban areas, respectively (12). Previous studies found that incident CVD and its mortality were associated with unhealthy lifestyle behaviors (13–15), overweight and obesity (16), emotional problem (17), cardiorespiratory fitness (18) and insomnia or poor sleep (19). In this regard, the progression of CVD may involve multiple risk factors acting together over a long-term period of time, suggesting that the underlying mechanisms of CVD, its risk factors and improved treatment remain a hot field of further research.

The contribution of chronic diseases to functional disabilities has been widely examined in previous studies. For example, a study conducted in seven countries with low and middle incomes showed that dementia made the largest contribution to functional disability, other substantial contributors were stroke, limb impairment, arthritis and so on (20). Similarly, a cross-sectional study conducted among community-dwelling oldest-old in Israel reported that stroke and heart attack were associated with functional limitations according to both ADL and IADL scales (21). Also, in a Chinese Longitudinal Healthy Longevity Survey, stroke or cerebrovascular disease and cognitive impairment were the strongest risk factors of functional disability among the elderly aged 80 years and over (22). However, the contribution of functional limitations to incident CVD among community-dwelling middle-aged and older adults is ambiguous. In addition, the association between functional disability and all-cause mortality has not been adequately explored among the Chinese population.

Therefore, this population-based prospective cohort study aimed to examine the association between functional limitations and incidence of CVD and all-cause mortality among the middle-aged and older adults in China. We hoped that this study could provide more valuable information for the prevention of incident CVD and death.

## Methods

### Study Population

Participants in this cohort study were from the China Health and Retirement Longitudinal Study (CHARLS), which is an ongoing nationally representative longitudinal study by the National School of Development at the Peking University. Details of the study design have been described elsewhere (23). In brief, a total of 17,708 participants were recruited by a multistage probability sampling procedure involving 150 counties or districts and 450 communities within 28 provinces of China at baseline in 2011 with a response rate of 80.5%. Data on sociodemographic and lifestyle behaviors factors and health-related information were collected by about 500 professional interviewers who worked in this field, using a face-to-face computer-assisted personal interview (CAPI) system. All participants were followed up biennially after the baseline survey. In this study, participants aged 50 years and more were included, those with CVD and have missing values on functional status at baseline were excluded, and those having no response regarding their CVD status during the follow-up period were also excluded. The CHARLS was approved by the Biomedical Ethics Review Committee of Peking University (IRB00001052-11015; IRB00001052-11014) and written informed consent was obtained from all participants.

### Measures

#### Assessment of Functional Limitations

Functional limitations at baseline were assessed by the Katz activities of daily living (ADL) scale (24) and the Lawton instrumental activities of daily living (IADL) scale (25). CHARLS asked respondents if they required assistance with any of the six ADLs (dressing, bathing, eating, getting into and out of bed, toileting and controlling urination and defecation) and with any of the five IADLs (preparing a hot meal, shopping for groceries, doing housework, taking medicines and managing money). Each item was divided into four responses as follows: (1) No, I do not have any difficulty, (2) I have difficulty but still can do it, (3) Yes, I have difficulty and need help, and (4) I cannot do it. Participants were scored 0 for responding “no difficulty”, (1) for responding “have difficulty but still can do it”, (2) for responding “have difficulty and need help”, and (3) for responding “cannot do it”. The summed score over the six ADL items served as the total score of the ADL scale for a subject, and this ranged from 0 to 18. The summed score over the five IADL items served as the total score of the IADL scale for a subject, and this ranged from 0 to 15. Participants who reported needing any help in any item (score of 2 or 3 in any item) were classified as having ADL or/and IADL limitations, thereafter called functional limitations (26).

#### Ascertainment of Incident CVD Events and Deaths

The study outcome was incident CVD and death. In accordance with previous studies (27, 28), incident CVD was assessed by asking the following standardized questions based on a self-reported physician's diagnosis: “Have you been diagnosed with a heart attack, coronary heart disease, angina, congestive heart failure, or other heart problems by a doctor?” or “Have you been diagnosed with a stroke by a doctor?”. Participants who reported heart disease or stroke during the follow-up period were defined as having incident CVD. The date of CVD diagnosis was recorded as between the date of last interview and the date of interview when an incident CVD was reported (27). The mortality status from all causes and death date were ascertained by interviewers in each visit. Furthermore, at every interview wave, interviews with earlier respondents were sought. If a respondent's death was reported, the CHARLS team identified a knowledgeable informant (typically a family member) and conducted exit interviews to obtain information about the death (29).

### Covariates

Information related to sociodemographic characteristics, health-related factors, depressive symptoms and social participation were collected by trained interviewers using a structured questionnaire at baseline. Precisely, sociodemographic information included age, gender, residential area (urban/rural), marital status and education level. Marital status was classified as married and unmarried (including separated, divorced, widowed and never married). Education level was classified as no formal education, primary school, middle or high school, and college or above. Health-related factors included self-reported smoking status (current/former/never), drinking status (current/former/never), sleep duration (<7h;7–7.9h and ≥8h) and nap duration (<30 min; 30–59 min and ≥60 min). Sleep duration was assessed using a single item, “During the past month, how many hours of actual sleep did you get at night (average hours for one night) (This may be shorter than the number of hours you spend in bed)?”. Nap duration was assessed by a single item, “During the past month, how long did you take a nap after lunch?”. Self-reported physician-diagnosed medical conditions included diabetes, hypertension and dyslipidemia. Use of medication for hypertension, diabetes and dyslipidemia was also assessed. Depressive symptoms in the past week were assessed at baseline using the 10-items Center for Epidemiologic Studies Depression Scale short form (CESD-10), participants with total score of 12 or higher were defined as having depressive symptoms (30). Participants not participating in any social activities over the last month (e.g. interacting with friends; playing chess or cards; attending a community sports, social or other club; participating in a community-related organization; participating in voluntary or charity work; and attending any educational or training courses) were classified as having low social participation (31). Moreover, anthropometric measurements including height, weight, and blood pressure (diastolic blood pressure and systolic blood pressure) were measured by a trained nurse following standard tools and procedures in CHARLS. Body mass index (BMI) was calculated according to the formula of kg/m^2^. Additionally, venous blood sample was collected in CHARLS participants and assayed for fasting plasma glucose (FPG), total cholesterol (TC), triglycerides (TG), high-density lipoprotein cholesterol (HDL-C), low-density lipoprotein cholesterol (LDL-C-), high-sensitivity C-reactive protein and glycated hemoglobin (HbA1c) (32).

### Statistical Analysis

Characteristics of the overall sample at baseline were summarized using mean and standard deviation (SD) for normally distributed continuous data, or median and interquartile range for skewed continuous data. Categorical data were summarized as counts and percentages. The chi-square test, student's *t*-test or Mann-Whitney U test were used to compare study sample characteristics at baseline between participants with and without functional limitations. Missing data items, 14.8% (1,631 of 11,013) in the baseline covariates were imputed using the multiple imputation of chained equations. Pooled results were reported based on five imputed data sets, which were created using command “mi estimate” in Stata statistical software version 16.0.

Person-time of follow-up for each participant was calculated from the date of the 2011 baseline survey to the dates of CVD diagnosis, death, loss to follow-up [820 of 11,013 (7.4%)], or end of follow-up (August 31, 2018), whichever came first. Incidence rate of CVD and death per 1,000 person-years were computed according to different functional status. The Cox proportional hazards model was used to assess the association between functional limitations and incident CVD and all-cause mortality. The effect size of this association was computed as the adjusted hazards ratios (HR) with 95% confidence interval (CI) in three models. That is, the association was adjusted for age and gender in model 1, and it was adjusted for age, gender, residential area, marital status, education, smoking, drinking, sleep duration, nap duration and depression symptoms in model 2. Finally, the variables adjusted for in model 2 plus history of diabetes, hypertension, dyslipidemia, use of medication for hypertension, dyslipidemia, diabetes and social participation were adjusted for the association in model 3. In addition, three-knotted restricted cubic spline regression was used to explore the potential non-linear association between functional limitation and CVD as well as all-cause mortality. To examine the possible interaction between functional status and other factors for incident CVD and deaths, subgroup analyses were conducted by stratification according to following factors: age (<60 or ≥60 years), gender, marital status, residential area, educational level, smoking, drinking, sleep duration, nap duration, depressive symptoms, social participation, diabetes (defined as FPG ≥ 126 mg/dL and/or HbA1c ≥ 6.5%, current use of antidiabetic medication, or self-reported history of diabetes), dyslipidemia (defined as TC ≥ 240 mg/dL, TG ≥ 150 mg/dL, LDL-C ≥ 160 mg/dL, HDL-C <40 mg/dL, current use of lipid-lowering medication, or self-reported history of dyslipidemia) and hypertension (defined as systolic blood pressure ≥ 140 mm Hg, diastolic blood pressure ≥ 90 mm Hg, current use of antihypertensive medication, or self-reported history of hypertension). The *P* values for interactions were calculated using interaction terms and likelihood ratio tests.

We conducted three sensitivity analyses as follows: (1) repeating all analysis using the complete data set (9,382 participants) without multiple imputations; (2) reporting the results after adjusting for BMI, blood pressure, TC, TG, HDL-C, LDL-C, HbA1c and C-reactive protein based on model 3 in the subpopulation of 5,388 participants; (3) using the Fine and Gray competing risk model to account for competing risks due to mortality when examining the association between functional limitation and incidence of CVD and death (33); and (4) repeating all analysis after excluding participants who had a CVD event within 3 years. Two-sided *P* < 0.05 was considered as statistically significant. All analyses were performed using Stata statistical software version 16.0 (Stata Corp, College Station, Texas, USA) and R statistical software version 4.0.3 (R Foundation).

## Results

A total of 17,708 CHARLS participants were available at baseline for sample selection. Of these, 3,730 were excluded for being younger than 50 years, 1,841 for having a heart disease at baseline, 425 for having stroke at baseline, 267 for having missing values on functional status, and 432 were excluded for having no response regarding their CVD during the follow-up period. Finally, 11,013 participants were included in this study for analysis. A comparison of baseline characteristics between the included participants and those not included in the analysis is shown in Supplementary Table S1.

Considering the sample included in this study for analysis, the mean (SD) age at baseline was 62.1 (8.6) years, proportion of men was 5,604 (50.9%) and the proportion of those living in rural areas was 6,997 (63.5%). Furthermore, 2,458 (22.4%) participants self-reported that they had hypertension and 17.6% of them had been using antihypertension medication. The prevalence of functional limitations was 14.8% (1630/11013) at baseline. Table 1 shows the characteristics of participants at baseline according to functional status. Univariate analysis showed that participants who reported functional limitations were more likely to be older, be female, be living in rural areas, have no formal education, have self-reported hypertension, have self-reported diabetes or dyslipidemia, use antihypertension medications, use antidiabetic or lower-lipid medication, never smoke and drink, have depressive symptoms, have low social participation, have shorter sleep duration and higher BMI.

**Table 1:** Characteristics of participants at baseline according to functional status.

**Characteristics**	**Total sample (*n =* 11,013)**	**functional limitations**		***P* value^**a**^**
		**Yes (*n =* 1,630)**	**No (*n =* 9,383)**	
Age, years	62.1 (8.6)	67.2 (10.3)	61.2 (7.9)	<0.001
Male	5,604 (50.9)	668 (41.0)	4,936 (52.6)	<0.001
Rural residence	6,997 (63.5)	1,184 (72.6)	5,813 (62.0)	<0.001
Married	9,408 (85.4)	1,231 (75.5)	8,177 (87.1)	<0.001
Education level^b^				
No formal education	5,687 (51.7)	1,190 (73.1)	4,497 (48.0)	<0.001
Primary school	2,326 (21.1)	239 (14.7)	2,087 (22.3)	
Middle or high school	2,801 (25.5)	189 (11.6)	2,612 (27.9)	
College and above	184 (1.7)	10 (0.6)	174 (1.9)	
Self-reported chronic conditions^b^				
Hypertension	2,458 (22.4)	462 (28.6)	1,996 (21.3)	<0.001
Diabetes	536 (4.9)	118 (7.3)	418 (4.5)	<0.001
Dyslipidemia	812 (7.5)	141 (8.9)	671 (7.3)	0.020
Use of medication^b^				
Antihypertension	1,925 (17.6)	381 (23.6)	1,544 (16.5)	<0.001
Antidiabetic	380 (3.5)	83 (5.1)	297 (3.2)	<0.001
Lipid-lowering medication	448 (4.1)	90 (5.7)	358 (3.9)	0.001
Smoking				
Never	6,717 (61.0)	1,087 (66.7)	5,630 (60.0)	<0.001
Former	929 (8.4)	153 (9.4)	776 (8.3)	
Current	3,367 (30.6)	390 (23.9)	2,977 (31.7)	
Drinking				
Never	7,396 (67.2)	1,185 (72.7)	6,211 (66.2)	<0.001
Former	705 (6.4)	151 (9.3)	554 (5.9)	
Current	2,912 (26.4)	294 (18.0)	2,618 (27.9)	
Depression symptoms^b^	2,684 (27.9)	682 (52.9)	2,002 (24.0)	<0.001
Low social participation^b^	5,310 (51.8)	918 (63.2)	4,392 (49.9)	<0.001
Sleep duration ^b^				
<7 h	5,247 (51.6)	832 (58.3)	4,415 (50.5)	<0.001
7–7.9 h	1,879 (18.5)	178 (12.5)	1,701 (19.5)	
≥8 h	3,043 (29.9)	418 (29.3)	2,625 (30.0)	
Nap duration^b^				
<30 min	4,994 (48.8)	696 (48.2)	4,298 (48.9)	0.119
30–59 min	895 (8.7)	109 (7.5)	786 (8.9)	
≥60 min	4,344 (42.5)	640 (44.3)	3,704 (42.1)	
Body mass index (kg/m^2^)^c^	22.96 (3.55)	22.64 (3.42)	23.00 (3.57)	0.010
Blood pressure, mm Hg^c^				
SBP	132.36 (22.67)	132.00 (21.55)	132.41 (22.83)	0.648
DBP	75.91 (11.89)	75.84 (11.85)	75.91 (11.90)	0.076
Metabolic biomarkers^c^				
FPG, mg/dL	110.35 (35.10)	110.55 (36.65)	110.33 (34.86)	0.876
TC, mg/dL	194.26 (38.56)	193.87 (39.58)	194.32 (38.41)	0.773
TG, mg/dL	129.92 (94.07)	126.55 (84.97)	130.43 (95.36)	0.307
HDL-C, mg/dL	51.71 (15.55)	52.44 (16.34)	51.60 (15.43)	0.200
LDL-C, mg/dL	117.06 (35.44)	116.24 (35.43)	117.18 (35.45)	0.511
HbA1c, %	5.28 (0.81)	5.28 (0.83)	5.27 (0.81)	0.928
C-reactive protein, mg/L	1.08 (1.68)	1.08 (1.65)	1.14 (2.13)	0.156

*Data were presented as n (%) or mean (standard deviation) or median (interquartile range)*.

a *P values were obtained according to the chi-square test, student's t-test or Mann-Whitney U-test*.

b *Missing values: 15 for education; 47 for hypertension; 82 for diabetes; 194 for dyslipidemia; 47 for antihypertension medication; 82 for antidiabetic medication; 194 for lipid-lowering medication; 1,365 for depressive symptoms; 753 for social participation; 844 for sleep duration; and 780 for nap duration*.

c *Measured in subgroups of 5,388 participants. SBP, systolic blood pressure; DBP, diastolic blood pressure; FPG, fasting plasma glucose; TC, total cholesterol; TG, triglycerides; HDL-C, high-density lipoprotein cholesterol; LDL-C, low-density lipoprotein cholesterol; HbA1c, glycated hemoglobin*.

During the follow-up period (mean follow-up period of 6.7 (1.2) years), 1,914 individuals reported incident CVD (heart disease: 1,377; and stroke: 695) and 1,182 individuals died. The incidence rate of CVD was 36.04 per 1000 person-years among the participants with functional limitations, and 25.97 per 1000 person-years among the participants without functional limitations. The incidence of death was 6.33 per 1,000 person-years among the participants without functional limitations, and 14.04 per 1,000 person-years among the participant with functional limitations. After adjusting for potential confounders, participants with functional limitations were associated with a 23% increased risk of incident CVD (HR, 1.23, 95%CI: 1.08, 1.39). Moreover, individuals with functional limitations were associated with a 44% increased risk of incident stroke (HR, 1.44, 95%CI: 1.17, 1.77). However, there was no significant association between functional limitations at baseline and incident heart disease (HR, 1.06, 95%CI: 0.91, 1.24). Also, participants with functional limitations were associated with a 63% increased risk of all-cause mortality after adjusting for potential confounders (HR, 1.63, 95%CI: 1.41, 1.89). The results were shown in Table 2.

**Table 2:** Incidence rate of CVD and all-cause mortality according to functional status.

**Outcome**	**Cases, No**.	**Incidence rate, per 1,000 person-years**	**HR (95%CI)**		
			**Model 1^**a**^**	**Model 2^**b**^**	**Model 3^**c**^**
CVD (*n* = 1,914)					
Functional limitation					
No	1,570	25.97	1.00 [Reference]	1.00 [Reference]	1.00 [Reference]
Yes	344	36.04	1.30 (1.15,1.47)	1.29 (1.14,1.47)	1.23 (1.08,1.39)
Heart disease (*n* = 1,377)					
Functional limitation					
No	1,153	18.23	1.00 [Reference]	1.00 [Reference]	1.00 [Reference]
Yes	224	33.34	1.13 (0.97,1.31)	1.12 (0.96,1.31)	1.06 (0.91,1.24)
Stroke (*n* = 695)					
Functional limitation					
No	553	8.83	1.00 [Reference]	1.00 [Reference]	1.00 [Reference]
Yes	142	14.29	1.56 (1.29,1.89)	1.54 (1.26,1.89)	1.44 (1.17,1.77)
All-cause mortality (*n* = 1,182)					
Functional limitation					
No	408	6.33	1.00 [Reference]	1.00 [Reference]	1.00 [Reference]
Yes	774	14.04	2.06 (1.81,2.34)	1.68 (1.45,1.94)	1.63 (1.41,1.89)

*HR, hazards ratio; CI, confidence interval; CVD, cardiovascular disease*.

a *adjusted for age and gender*.

b *adjusted for age, gender, residential area, marital status, education level, smoking, drinking, sleep duration, nap duration and depression symptoms*.

c *adjusted for variables adjusted for in model 2 plus history of diabetes, hypertension, dyslipidemia, use of medication for hypertension, use of medication for dyslipidemia, use of medication for diabetes and social participation*.

**Figure 1:**
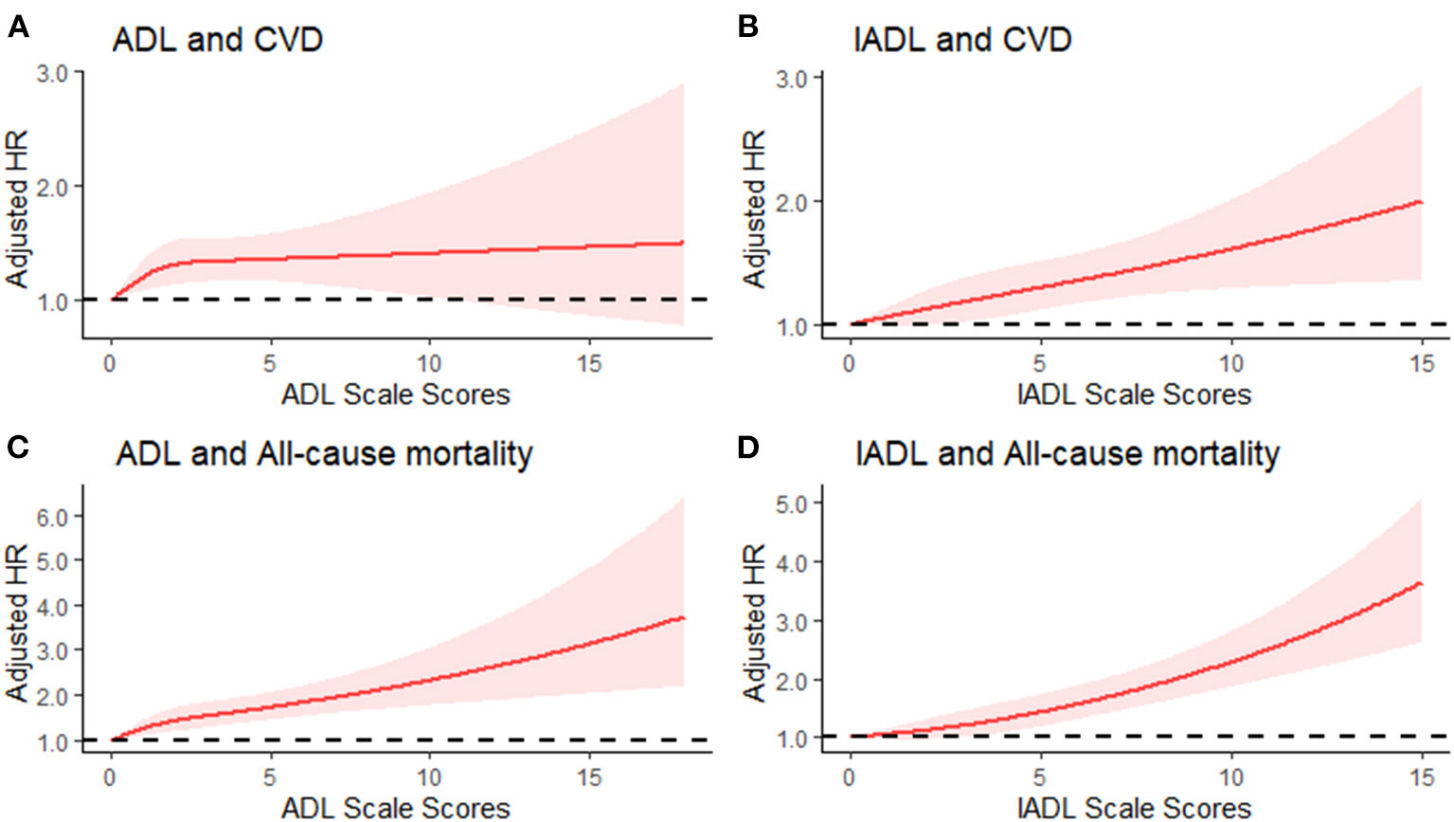
Adjusted Hazard Ratio (HRs) of cardiovascular disease and all-cause mortality according to ADL and IADL scale score. **(A)** Adjusted HRs for association between ADL score and CVD; **(B)** Adjusted HRs for association between IADL score and CVD; **(C)** Adjusted HRs for association between ADL score and all-cause mortality; **(D)** Adjusted HRs for association between IADL score and all-cause mortality.

Similar results were obtained when subgroup analyses were conducted between ADL or IADL limitations and incident CVD and all-cause mortality (Supplementary Table S2). In this regard, participants with ADL limitations were associated with a 25 and 105% increased risk of CVD (HR, 1.25, 95%CI: 1.04, 1.50) and all-cause mortality (HR, 2.05, 95%CI: 1.72, 2.46), respectively. On the other hand, individuals with IADL limitations were associated with a 23 and 66% increased risk of CVD (HR, 1.23, 95%CI: 1.08, 1.41) and all-cause mortality (HR, 1.66, 95%CI: 1.43, 1.93), respectively. Further, results of the 3-knotted restricted cubic spline regression model indicated a linear association between ADL and CVD (*P* = 0.474) as well as between IADL and CVD (*P* = 0.751). Similarly, there was a linear association between IADL and all-cause mortality (*P* = 0.494), but there was a positive nonlinear association between ADL and all-cause mortality (*P* = 0.044). The results are presented in Figure 1.

**Figure 2:**
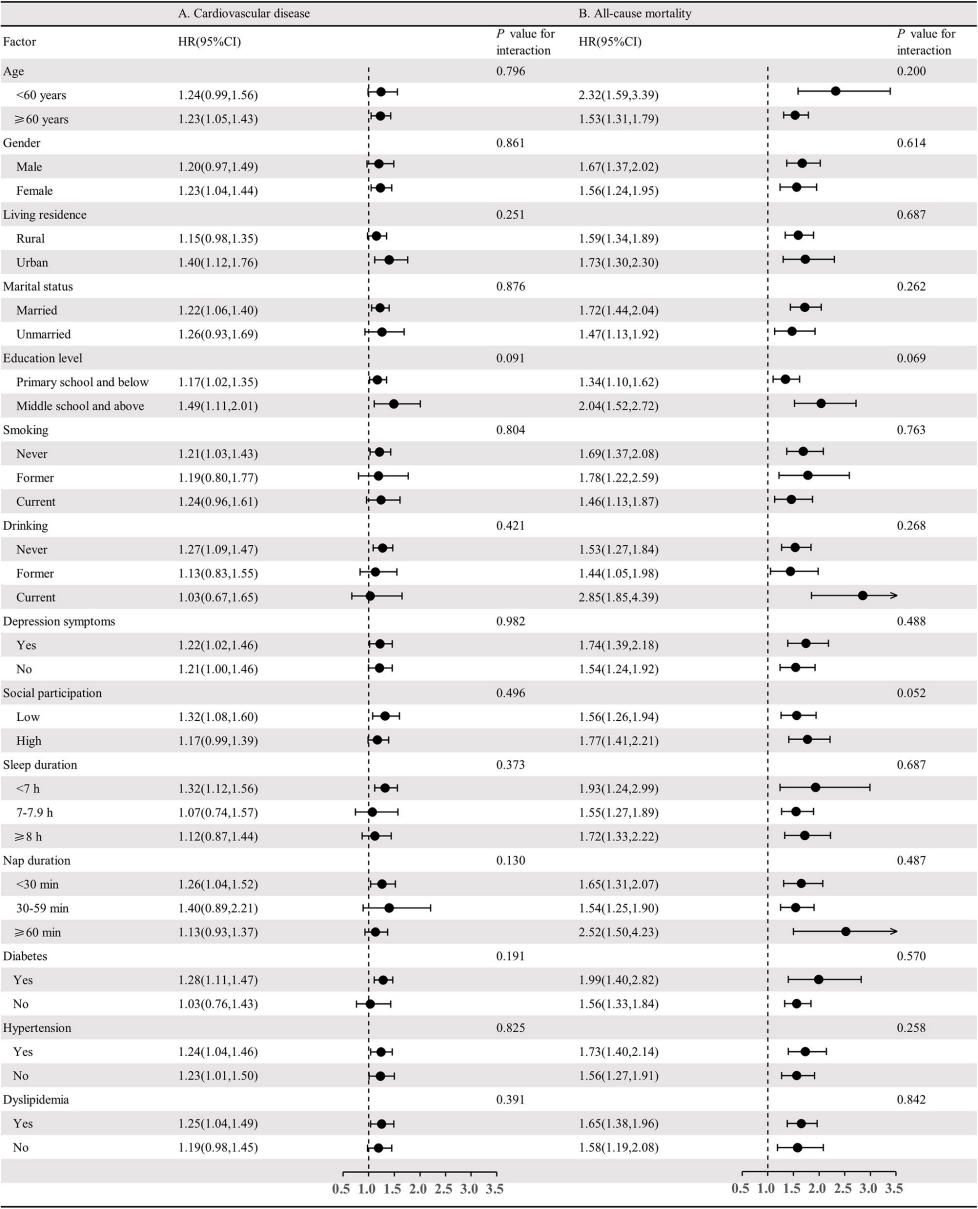
Association between functional limitation and risk of cardiovascular disease and all-cause mortality stratified by different factors. **(A)** Adjusted HRs for association between functional limitation and CVD; **(B)** Adjusted HRs for association between functional limitation and all-cause mortality.

Also, results on the possible interaction between functional status and other factors for incident CVD and all-cause mortality showed that there was no significant interaction between functional limitations and potential risk factors for incident CVD and all-cause mortality (Figure 2 shows the details). Similarly, complete data analyses for a subpopulation of 9,382 participants (Supplementary Table S3) did not change the earlier found results on the association between functional limitations and incident CVD and all-cause mortality. Likewise, these results did not significantly change after adjusting for BMI, blood pressure, FPG, TG, TC, HDL-C, LDL-C, HbA1c and C-recreative protein in a subpopulation of 5,388 participants (Supplementary Table S4). Sensitivity analysis excluding participants who had a CVD event within 3 years of follow-up showed that the associations between functional limitations and the risk of CVD and all-cause mortality were unchanged (Supplementary Table S5). In addition, people with functional limitations were associated with a 20% increased risk for incident CVD, after adjusting for potential confounders, when using the Fine and Gray model with death as competing risk event (Supplementary Table S6).

## Discussions

Based on a nationally representative sample, we longitudinally examined the association between functional limitations and incident CVD and all-cause mortality, among the Chinese adults aged at least 50 in China, during 7 years of follow-up. The prevalence of functional limitations was 14.80% at baseline. Moreover, functional limitations were associated with a 23 and 63% increased risk of CVD and all-cause mortality, respectively.

There has been growing evidence suggesting that the presence of functional limitations is associated with increased risk of CVD and mortality. For example, the Cardiovascular Health Study demonstrated that limitations in ADL modified the association of blood pressure with CVDs and deaths. That is, among people with ADL limitations, a high systolic blood pressure remained associated with higher risk of incident CVD, whereas the association of diastolic blood pressure with CVD was inverted (34). A prospective cohort study conducted by Tsuji et al. demonstrated that the Japanese adults aged at least 65 with ADL limitations were significantly associated with increased risk of stroke and its mortality, and their limitation in ambulatory activity was significantly associated with increased risk of heart disease mortality (35). Similarly, a large cohort study conducted in the United States demonstrated that functional limitations were powerful independent predictors of mortality among older participants aged at least 80 (36). Moreover, in the older Dutch population, a 15-year follow-up cohort study showed that functional limitations were significant predictors of all-cause mortality (37).

Although the association between functional limitations and incidence of CVD and all-cause mortality was statistically significant in this study, the hazards ratio continuously declined from model 1 to model 3, suggesting that lifestyle behaviors, emotional problems, chronic conditions, and social participation might have played a significant role in mediating the association between functional limitations and the risk of CVD and all-cause mortality. However, the underlying mechanisms in such a “black box” are complex and multifactorial. In terms of biological mechanisms, the functional limitations tended to be the consequence of physiological changes associated with aging (38). Using three clinical-biomarker-algorithm methods, older adults with more advanced biological aging reported dependence in more ADLs and IADLs, and were at increased risk of death (39). Growing evidence has also supported the hypothesis that aging is accompanied by impairments in heart (40), vascular structure and functioning (41), which may further lead to increase in the risk of CVD and mortality (42). Thus, functional limitations and CVD are highly interconnected and may share common biological pathways. Furthermore, from a social psychological point of view, disability in late life has been associated with increased dependence and loss of autonomy. Functional limitations among the elderly have also often been accompanied with reduced likelihood of social activities and social contact (43, 44). Additionally, functional limitations may be associated with worse relationship with offspring due to the fact that increased dependence of parents with functional limitations on help may go beyond the routine support; hence may cause more friction between them and their children or caregivers (45). Also, older adults with physical functional limitations were at higher risk of depressive symptoms, anxiety symptoms and suicidal ideation (46, 47). Functional limitations are often accompanied with depression and may evoke inflammation, platelet activation and thrombosis, and autonomic nerve dysfunction (48). All of these were potential risk factors for stroke (49) and myocardial ischemia (50), hence may increase the risk of mortality (51). Undoubtedly this is an important pathway to the delay or development of CVD and death. As regards behavioral factors, people with functional limitations in ADLs or IADLs were more likely to report smoking (52), lower level of physical activity (53), and lower sleep efficiency or irregular sleep (54), which in turn are risk factors for CVD and all-cause mortality (55, 56).

Further, the substantial prevalence of functional limitations, among the middle-aged and older adults in this study, underscores the importance of interventions aimed at prevention and control of CVD incidences in this group. These may include physical therapy or physical activity interventions for improving underlying impairments in physical abilities (57).

The main strength of this study is that it used a representative sample, and examined the outcome variables of interest in a considerably longer period of followed-up time (7 years). Therefore, adequate information was collected for the purpose of this study. Besides, a wide range of potential confounders were taken into account to control confounding bias and clearly understand the association between functional limitations and incident CVD and all-cause mortality. Also, the application of sensitivity analyses presented a more complete picture of the association between functional limitations and incident CVD and death. Specifically, sensitivity analyses showed that the result were stable and hence more reliable. Despite the foregoing strengths of this study, several potential limitations need to be acknowledged. First, the occurrence of CVD and death were self-reported because medical records were not available in the CHARLS. However, there has been a high agreement between self-reports and medical records of the elderly with cardiovascular diseases (58). Second, functional limitations were self-reported, which could exaggerate its prevalence in the sample. However, the ADL scale and IADL scale are the most frequently used self-reported questionnaires in the measurement of disability in the elderly (59). Third, there may be a bidirectional effect between functional limitations and CVD, but the subsequent impact of CVD on the functional limitations was not clarified in this study. Fourth, some potential covariates of the association between functional limitations and CVD and all-cause mortality, such as income (60), social isolation (61) and physical activity (62) were not considered in this study due to dataset limitations. Finally, only participants from China were involved in this study, thus the findings may not apply to populations of other countries.

## Conclusions

This study found that functional limitations among the Chinese middle-aged and older adults were significantly associated with incident CVD and all-cause mortality. Prevention and control of functional limitations maybe a potential way to reduce the risk of CVD and death.

## Data Availability Statement

CHARLS data are available at http://charls.pku.edu.cn/pages/data/111/zh-cn.html. The original contributions presented in the study are included in the article/Supplementary Material, further inquiries can be directed to the corresponding author/s.

## Ethics Statement

The studies involving human participants were reviewed and approved by Biomedical Ethics Review Committee of Peking University. The patients/participants provided their written informed consent to participate in this study.

## Author Contributions

ZH and HX designed the study. ZH and BZ wrote and revised the manuscript. FZ conducted the analyses and prepared Figure 2. AK edited the manuscript. All authors reviewed the manuscript.

## Conflict of Interest

The authors declare that the research was conducted in the absence of any commercial or financial relationships that could be construed as a potential conflict of interest.

## Publisher's Note

All claims expressed in this article are solely those of the authors and do not necessarily represent those of their affiliated organizations, or those of the publisher, the editors and the reviewers. Any product that may be evaluated in this article, or claim that may be made by its manufacturer, is not guaranteed or endorsed by the publisher.
