# Supplementary material for: Association Between Functional Limitations and Incident Cardiovascular Diseases and All-Cause Mortality Among the Middle-Aged and Older Adults in China: A Population-Based Prospective Cohort Study
Source: Front Public Health. 2022 Feb 11;10:751985. doi: 10.3389/fpubh.2022.751985 (PMC8873112; doi:10.3389/fpubh.2022.751985)
Supplement: Supplementary file 1 [file Data_Sheet_1.docx]

**Supplement material**

Table S1 Baseline Characteristics between participants included and not included

Table S2 Association between ADL or IADL limitation and incident CVD and all-cause mortality

Table S3 Association between functional limitation and incident cardiovascular disease and all-cause mortality among subpopulation of 9382 participants with complete data

Table S4 Association between functional limitation and incident cardiovascular disease and all- cause mortality among subpopulation of 5388 participants with metabolic biomarkers, BMI and blood pressure

Table S5 Association of functional limitation With Cardiovascular Diseases and all-cause mortality after excluded CVD cases in first 3 years follow-up

Table S6 Association of functional limitation With Cardiovascular Diseases by Competing Risk Analysis

Table S1 Baseline Characteristics between participants included and not included

| Characteristics | Excluded(n=6695) | Included(n=11013) | *P* value ^a^ |
| --- | --- | --- | --- |
| Age, years, mean(SD) | 54.0(10.5) | 62.1(8.6) | <0.001 |
| Male | 2867(42.9) | 5604(50.9) | <0.001 |
| Rural residence | 3540(52.9) | 6997(63.5) | <0.001 |
| Married | 6009(90.2) | 9408(85.4) | <0.001 |
| Education level |  |  |  |
| No formal education | 1269(19.1) | 3534(32.1) | <0.001 |
| Primary school | 2473(37.2) | 4479(40.7) |  |
| Middle or high school | 2668(40.1) | 2801(25.5) |  |
| College and above | 245(3.7) | 184(1.7) |  |
| Self-reported chronic conditions |  |  |  |
| Hypertension | 1826(28.1) | 2458(22.4) | <0.001 |
| Diabetes | 457(7.1) | 536(4.9) | <0.001 |
| Dyslipidemia | 783(12.2) | 812(7.5) | <0.001 |
| Use of medication ^b^ |  |  |  |
| Antihypertension | 1487(22.2) | 1925(17.6) | 0.009 |
| Antidiabetic | 332(5.0) | 380(3.5) | 0.284 |
| Lipid-lowering medication | 463(6.9) | 448(4.1) | 0.087 |
| Smoking |  |  |  |
| Never | 4677(69.9) | 6717(61.0) | <0.001 |
| Former | 516(7.7) | 929(8.4) |  |
| Current | 1499(22.4) | 3367(30.6) |  |
| Drinking |  |  |  |
| Never | 4902(73.3) | 7396(67.2) | <0.001 |
| Former | 361(5.4) | 705(6.4) |  |
| Current | 1429(21.4) | 2912(26.4) |  |
| Depression symptoms | 1603(28.4) | 2684(27.9) | 0.482 |
| Low social participation | 2785(46.7) | 5310(51.8) | <0.001 |
| Sleep duration |  |  |  |
| <7 h | 2857(48.3) | 5247(51.6) | <0.001 |
| 7-7.9h | 1257(21.2) | 1879(18.5) |  |
| ≥8h | 1806(30.5) | 3043(29.9) |  |
| Nap duration |  |  |  |
| <30 min | 2873(48.3) | 4994(48.8) | 0.119 |
| 30-59 min | 578(9.7) | 895(8.7) |  |
| ≥60 min | 2499(42.0) | 4344(42.5) |  |

Data were present as n(%) or mean(SD).

^a^ *P*-value was obtained by the Chi-square test.

Table S2 Association between ADL or IADL limitation and incident CVD and all-cause mortality

| Outcome | HR(95%CI) | | |
| --- | --- | --- | --- |
|  | Model 1^a^ | Model 2^b^ | Model 3^c^ |
| Cardiovascular disease(n=1914) |  |  |  |
| ADL limitation |  |  |  |
| No | Reference | Reference | Reference |
| Yes | 1.46(1.22,1.74) | 1.33(1.09,1.62) | 1.25(1.04,1.50) |
| IADL limitation |  |  |  |
| No | Reference | Reference | Reference |
| Yes | 1.30(1.14,1.47) | 1.29(1.13,1.48) | 1.23(1.08,1.41) |
| Heart disease(n=1377) |  |  |  |
| ADL limitation |  |  |  |
| No | Reference | Reference | Reference |
| Yes | 1.08(0.81,1.32) | 1.04(0.75,1.26) | 1.02(0.68,1.14) |
| IADL limitation |  |  |  |
| No | Reference | Reference | Reference |
| Yes | 1.14(0.98,1.33) | 1.14(0.97,1.34) | 1.08(0.92,1.27) |
| Stroke(n=695) |  |  |  |
| ADL limitation |  |  |  |
| No | Reference | Reference | Reference |
| Yes | 2.25(1.75,2.90) | 1.99(1.51,2.63) | 1.77(1.34,2.34) |
| IADL limitation |  |  |  |
| No | Reference | Reference | Reference |
| Yes | 1.60(1.31,1.95) | 1.59(1.28,1.96) | 1.49(1.20,1.84) |
| All-cause mortality(n=1182) |  |  |  |
| ADL limitation |  |  |  |
| No | Reference | Reference | Reference |
| Yes | 2.51(2.15,2.93) | 2.15(1.80,2.57 | 2.05(1.72,2.46) |
| IADL limitation |  |  |  |
| No | Reference | Reference | Reference |
| Yes | 2.12(1.86,2.41) | 1.71(1.47,1.99) | 1.66(1.43,1.93) |

^a^ adjusted for age and gender.

^b^ adjusted for age, gender, living residence, marital status, education, smoking, drinking, sleep duration, nap duration and depression symptoms.

^c^ adjusted for variables in model 2 plus history of diabetes, hypertension, dyslipidemia, and use of medication for hypertension, dyslipidemia, diabetes and social participation.

Table S3 Association between functional limitation and incident cardiovascular disease and all-cause mortality among subpopulation of 9382 participants with complete data

| Outcome | HR(95%CI) | | |
| --- | --- | --- | --- |
|  | Model 1^a^ | Model 2^b^ | Model 3^c^ |
| Cardiovascular disease(n=1659) |  |  |  |
| Functional limitation |  |  |  |
| No | Reference | Reference | Reference |
| Yes | 1.34(1.18,1.53) | 1.32(1.15,1.52) | 1.25(1.09,1.43) |
| Heart disease(n=1201) |  |  |  |
| Functional limitation |  |  |  |
| No | Reference | Reference | Reference |
| Yes | 1.18(1.01,1.39) | 1.15(0.97,1.35) | 1.08(0.92,1.28) |
| Stroke(n=595) |  |  |  |
| Functional limitation |  |  |  |
| No | Reference | Reference | Reference |
| Yes | 1.64(1.33,2.02) | 1.63(1.31,2.02) | 1.51(1.21,1.88) |
| All-cause mortality(n=917) |  |  |  |
| Functional limitation |  |  |  |
| No | Reference | Reference | Reference |
| Yes | 1.83(1.57,2.13) | 1.61(1.37,1.88) | 1.57(1.34,1.84) |

^a^ adjusted for age and gender.

^b^ adjusted for age, gender, living residence, marital status, education, smoking, drinking, sleep duration, nap duration and depression symptoms.

^c^ adjusted for variables in model 2 plus history of diabetes, hypertension, dyslipidemia, and use of medication for hypertension, dyslipidemia, diabetes and social participation.

Table S4 Association between functional limitation and incident cardiovascular disease and all- cause mortality among subpopulation of 5388 participants with metabolic biomarkers, BMI and blood pressure

| Outcome | HR(95%CI) | | | |
| --- | --- | --- | --- | --- |
|  | Model 1^a^ | Model 2^b^ | Model 3^c^ | Model 4^d^ |
| Cardiovascular disease(n=997) |  |  |  |  |
| Functional limitation |  |  |  |  |
| No | Reference | Reference | Reference | Reference |
| Yes | 1.42(1.20,1.68) | 1.39(1.17,1.65) | 1.31(1.10,1.56) | 1.31(1.10,1.56) |
| Heart disease(n=716) |  |  |  |  |
| Functional limitation |  |  |  |  |
| No | Reference | Reference | Reference | Reference |
| Yes | 1.17(0.95,1.44) | 1.13(0.92,1.40) | 1.07(0.86,1.32) | 1.07(0.86,1.33) |
| Stroke(n=366) |  |  |  |  |
| Functional limitation |  |  |  |  |
| No | Reference | Reference | Reference | Reference |
| Yes | 1.74(1.34,2.26) | 1.70(1.30,2.23) | 1.56(1.19,2.04) | 1.57(1.20,2.07) |
| All-cause mortality(n=488) |  |  |  |  |
| Functional limitation |  |  |  |  |
| No | Reference | Reference | Reference | Reference |
| Yes | 1.85(1.50,2.29) | 1.67(1.34,2.08) | 1.63(1.31,2.03) | 1.59(1.27,1.98) |

^a^ adjusted for age and gender.

^b^ adjusted for age, gender, living residence, marital status, education, smoking, drinking, sleep duration, nap duration and depression symptoms.

^c^ adjusted for variables in model 2 plus history of diabetes, hypertension, dyslipidemia, and use of medication for hypertension, dyslipidemia, diabetes and social participation.

^d^ adjusted for variables in model 3 plus BMI, blood pressure, FPG,TG,TC,HDL-C,LDL-C,HbA1c and C-recreative protein.

Table S5 Association of functional limitation With Cardiovascular Diseases and all-cause mortality after excluded CVD cases in first 3 years follow-up

| Outcome | HR(95%CI) | | |
| --- | --- | --- | --- |
|  | Model 1^a^ | Model 2^b^ | Model 3^c^ |
| Cardiovascular disease(n=1520) |  |  |  |
| Functional limitation |  |  |  |
| No | Reference | Reference | Reference |
| Yes | 1.33(1.16,1.53) | 1.33(1.16,1.53) | 1.25(1.09,1.44) |
| Heart disease(n=1043) |  |  |  |
| Functional limitation |  |  |  |
| No | Reference | Reference | Reference |
| Yes | 1.11(0.93,1.32) | 1.09(0.91,1.30) | 1.02(0.86,1.22) |
| Stroke(n=579) |  |  |  |
| Functional limitation |  |  |  |
| No | Reference | Reference | Reference |
| Yes | 1.63(1.32,2.01) | 1.66(1.34,2.06) | 1.55(1.25,1.92) |
| All-cause mortality (n=907) |  |  |  |
| Functional limitation |  |  |  |
| No | Reference | Reference | Reference |
| Yes | 1.89(1.63,2.20) | 1.75(1.50,2.04) | 1.71(1.47,1.99) |

^a^ adjusted for age and gender.

^b^ adjusted for age, gender, living residence, marital status, education, smoking, drinking, sleep duration, nap duration and depression symptoms.

^c^ adjusted for variables in model 2 plus history of diabetes, hypertension, dyslipidemia, and use of medication for hypertension, dyslipidemia, diabetes and social participation.

Table S6 Association of functional limitation With Cardiovascular Diseases by Competing Risk Analysis

| Outcome | HR(95%CI) | | |
| --- | --- | --- | --- |
|  | Model 1^a^ | Model 2^b^ | Model 3^c^ |
| Cardiovascular disease |  |  |  |
| Functional limitation |  |  |  |
| No | Reference | Reference | Reference |
| Yes | 1.28(1.13,1.46) | 1.26(1.11,1.44) | 1.20(1.05,1.37) |
| Heart disease |  |  |  |
| Functional limitation |  |  |  |
| No | Reference | Reference | Reference |
| Yes | 1.14(0.97,1.33) | 1.10(0.94,1.30) | 1.05(0.89,1.23) |
| Stroke |  |  |  |
| Functional limitation |  |  |  |
| No | Reference | Reference | Reference |
| Yes | 1.54(1.25,1.91) | 1.54(1.24,1.92) | 1.43(1.15,1.78) |

^a^ adjusted for age and gender.

^b^ adjusted for age, gender, living residence, marital status, education, smoking, drinking, sleep duration, nap duration and depression symptoms.

^c^ adjusted for variables in model 2 plus history of diabetes, hypertension, dyslipidemia, and use of medication for hypertension, dyslipidemia, diabetes and social participation.
